# Pyruvate-Enriched Oral Rehydration Solution Improves Glucometabolic Disorders in the Kidneys of Diabetic *db/db* Mice

**DOI:** 10.1155/2020/2817972

**Published:** 2020-09-24

**Authors:** Xiao Meng Zhang, Hao Deng, Jin Dong Tong, Yi Zhen Wang, Xu Chao Ning, Xiu Hong Yang, Fang Qiang Zhou, Hui Min Jin

**Affiliations:** ^1^Department of Nephrology, Pudong Hospital, Shanghai Medical School, Fudan University, 2800 Gong Wei Road, Shanghai, China; ^2^Division of Vascular surgery, Pudong Hospital, Shanghai Medical School, Fudan University, 2800 Gong Wei Road, Shanghai, China; ^3^Department of Clinical Medicine, Affiliated Hospital of Qingdao University, 16 Jiang Su Road, Qingdao, Shandong, China; ^4^Shanghai Sandai Pharmaceutical R&D Co., Ltd., Pudong, Shanghai, China

## Abstract

Diabetes is prevalent worldwide, but ideally intensive therapeutic strategy in clinical diabetes and diabetic nephropathy (DN) is still lack. Pyruvate is protective from glucometabolic disturbances and kidney dysfunction in various pathogenic insults. Present studies focused on oral pyruvate effects on diabetes status and DN with 0.35% pyruvate in pyruvate-enriched oral rehydration solution (Pyr-ORS) and 1% pyruvate as drinking water for 8 weeks, using the model of diabetic *db/db* mice. Both Pyr-ORS and 1% pyruvate showed comparable therapeutic effectiveness with controls of body weight and blood sugar, increases of blood insulin levels, and improvement of renal function and pathological changes. Aberrant key enzyme activities in glucometabolic pathways, AR, PK, and PDK/PDH, were also restored; indexes of oxidative stress and inflammation, NAD^+^/NADH ratio, and AGEs in the kidneys were mostly significantly preserved after pyruvate treatments. We concluded that oral pyruvate delayed DN progression in *db/db* mice and the modified Pyr-ORS formula might be an ideal novel therapeutic drink in clinical prevention and treatment of type 2 diabetes and DN.

## 1. Introduction

Diabetes has become one of the most severe chronic diseases worldwide. According to 2017 statistics, diabetes mellitus (DM) patients are about 425 million in the world, which is about three times the number of diabetic patients in 2000 [1]. Diabetic nephropathy (DN) develops in over 30% of diabetic patients and is the leading cause of chronic kidney disease and end-stage renal disease with a high mortality in westernized countries [2].

Accumulating studies demonstrated that disturbances of glucose metabolic pathways, particularly, pyruvate kinase (PK M2) depression and the Warburg phenomenon, glomerular microvascular endothelial mitochondrial dysfunction, and endoplasmic reticulum stress in renal proximal tubular epithelial cells all stimulated by hyperglycemia (HG) play crucial roles in DN onset and progression in diabetic rodent models and patients [3–7]. Specifically, renal hypoxia, oxidative stress, inflammation, and advanced glycation end products (AGEs) are simultaneously participated in the process, leading to a vicious circle in DN exacerbation [7–10]. However, underlying molecular and metabolic mechanisms of DN initiation and progression are still not fully illustrated.

Numerous studies convincingly demonstrated that pyruvate was protective of glucose metabolism in various pathogenic insults [11–18]. Since 1990s, studies have shown that pyruvate displays the valuable protection against cellular aberrant glucometabolic pathways and redox status in HG status and diabetic cataract and retinopathy in animals and human cells [11, 12]. It has been revealed that pyruvate holds unique pleiotropic biological and pharmacological properties: increase of hypoxia tolerance, correction of glucometabolic disturbances and acid-base unbalance, action of antioxidative stress/inflammation, protection of mitochondria, and inhibition of cellular apoptosis [13–15]. Therefore, pyruvate robustly preserves multiorgan function, of which the kidney protection from oxidative stress, hypoxia/ischemia reperfusion, and poisoning injuries has been explicitly illustrated [16–18]. Further, oral 1-3% pyruvate apparently improved diabetic cataract and retinopathy, and pyruvate has been known to prevent or attenuate as well as delay AGE formation in rodent diabetic cataract lens [19, 20]. However, ingestion of pure pyruvate products does not benefit in humans (see below). In this terms, oral or enteral pyruvate (0.35%) in pyruvate-enriched, sodium-, glucose-containing oral rehydration solution (Pyr-ORS) has recently shown unequivocally beneficial in organ metabolism and function, including kidney, and significantly increases survival, relative to World Health Organization-guided ORS (WHO-ORS), in animals subjected to severe injuries [21, 22].

On this basis, the present study was undertaken to focus on effects of pyruvate-enriched fluid, i.e., Pyr-ORS (containing 0.35% pyruvate and 1.35% glucose) and pure 1% pyruvate as drinking water on type 2 diabetes, mainly DN, in diabetic *db/db* mice. As a preliminary descriptive study, results hereby ascertain that oral pyruvate is powerfully beneficial in diabetic *db/db* mice and first provide a possibility of the novel strategy with Pyr-ORS to improve glucometabolic profile in prevention and treatment of clinical type 2 diabetes and its organ complications including DN in a large population.

## 2. Materials and Methods

The experiment was conducted in compliance with the Guide for Care and Use of Laboratory Animals of the National Research Council of China, Beijing, China. All experimental protocols were reviewed and approved by the Ethics Committee of the Pudong Hospital, Shanghai Medical School, Fudan University, Shanghai, China.

### 2.1. Animal and Experimental Grouping

C57BLKS/J mice and diabetes mellitus (DM) C57BL/6 *db*/*db* mice (male, 6 weeks old) were purchased from Nanjing Biomedical Research Institute, Nanjing, China, and housed in plastic cages with a controlled temperature of 23-26°C, humidity of 50-55%, and a 12 h light/dark cycle in the specific pathogen free room (4 mice per cage). After acclimatized for 4 weeks, the DM C57BL/6 *db*/*db* mice were confirmed by the symptoms of hyperglycemia, polyphagia, polydipsia, and polyuria. Only those animals with fasting plasma glucose higher than 11.1 mmol/l (blood drawn from tail vein; glucose tested by Roche blood glucose meter) were selected as the diabetic model for the following experiments. A total of 24 animals at the age of 10 weeks old, including 6 C57BLKS/J normal nontreated mice as the normal group (group Nor), were selected, and 18 *db*/*db* DM mice were randomly divided into three groups with 6 mice in each. The control group (group Con) was DM nontreated mice. The pyruvate group (group Pyr) was DM mice drunk distilled water containing 1% sodium pyruvate (NaPyr), while the Pyr-ORS group (group Pyr-ORS) was DM mice given distilled water containing 0.35% NaPyr in oral rehydration salts (ORS). Groups Nor and Cor regularly fed experimental water. All the mice in four groups had free access to normal experimental diet with fluids mentioned above, respectively. The water consumption was above 10 ml/day by the weight of the water bottle each mouse. Body and kidney weights, fasting blood glucose and insulin concentrations, and 24-hour volume urine in a metabolic cage were collected and measured at the ages of 10 and 18 weeks old each mouse.

After 8 weeks, at the age of 18 weeks old, all animals were euthanized by an i.p. injection of pentobarbital sodium (200 mg/kg), and 1.3 ml of blood samples were collected by cardiac puncture each mouse, then centrifuged at 4000 r/min for 10 minutes to separate plasma, which were frozen at -80°C. The kidney was quickly removed from each mouse and frozen at -80°C. Pyr-ORS was fleshly prepared in the laboratory with 0.35% NaPyr, 0.2% NaCl, 0.15% Kcl, and 1.35% glucose anhydrate with 247 mOsm/l [23]. Sodium pyruvate was obtained from Thermo Fisher (Gibco; Thermo Fisher Scientific, Inc., Waltham, MA, USA).

### 2.2. Enzyme-Linked Immunosorbent Assay (ELISA)

Mouse kidney homogenates were individually prepared for detecting the levels of pyruvate dehydrogenase (PDH), including nonphosphorylated active pyruvate dehydrogenase (PDHa), total pyruvate dehydrogenase (PDHt) and pyruvate kinase (PK) (Yili Biology Technology, Shanghai, China); oxidized/reduced nicotinamide adenine dinucleotides (NAD^+^/NADH), Cystatin C (Cys-C), and AGEs (Jiancheng Bioengineering Institute, Nanjing, China); and insulin, lactate dehydrogenase (LDH), and radical oxygen species (ROS) (Yili Biology Technology, Shanghai, China), using respective ELISA kit following protocols. The levels of serum creatinine (Cr) and BUN and superoxide dismutase (SOD), catalase (CAT), malondialdehyde (MDA), glutathione peroxidase (GSH-PX), fructose, and sorbitol in kidney homogenates were detected; 24-hour urinary protein and neutrophil gelatinase-associated lipocalin (NGAL) were also measured with respective commercial assay kits (Nanjing Jiancheng, Nanjing, China).

The detection method of ROS was briefly as follows: diluted the sample and placed it in the plate, by adding the enzyme-labeled antibody, then the substrate solution for the color reaction, and finally, terminated the reaction by adding the stop solution. The OD value was measured with an enzyme mark instrument (infinite m200pro TECAN, Switzerland). The NAD^+^/NADH detection was briefly that tissues were lysed and heated with acidic/alkalic extracts to obtain NAD^+^ and NADH, then mixed with respective reagents and incubated at 80°C for 60 min in protection from light. The OD value was measured at a wavelength of 570 nm. The ratio was obtained through a specific calculation formula provided by the assay kit.

### 2.3. Western Blotting

The proteins used for Western blotting were extracted using lysis buffer (Inventbiotechnologies, INC, China), separated by the sodium dodecyl sulfate-polyacrylamide gel electrophoresis (SDS-PAGE), and transferred onto polyvinylidene difluoride (PVDF) membrane (Millipore, Bedford, MA, USA). The membrane carrying protein bands was blocked in Tris-buffered saline with Tween (TBST) with 5% skim milk for 1 h and incubated with primary antibodies (Proteintech Group, Inc., IL, USA) at 4°C overnight. After washing, the membrane was incubated with secondary antibodies marked by horseradish peroxidase for 1 h at room temperature. Secondary antibodies (Proteintech Group, Inc., IL, USA) included goat anti-rabbit IgG and goat anti-mouse IgG. After washing, the membrane carrying blots and antibodies was incubated with Immobilon Western Chemiluminescent HRP Substrate (Millipore). Signals of proteins were captured, using a Bio-Rad ChemiDoc™ XRS system (Bio-Rad, Hercules, CA, USA). Data were quantified by the Quantity One software (Bio-Rad). Primary antibodies against aldose reductase (AR) were purchased from GeneTex (CA, USA); those against PDH, PDHA1, and pyruvate dehydrogenase kinas (PDK) were purchased from Cell Signaling Technology (CST, Beverly, MA, USA); antibodies against PDK and PDHA1 were obtained from Abways Technology (Abways Technology Inc, Shanghai, China).

### 2.4. Immunohistochemistry Analysis

For the immunohistochemistry procedure for renal PDH, paraffin-embedded sections were prepared, dewaxed, and repaired using ethylenediaminetetraacetic acid (EDTA) antigen retrieval buffers. Next, the sections were incubated with the antibody (CST, Beverly, MA, USA) against PDH in PBS containing 3% bovine serum albumin (BSA) overnight at 4°C. The samples were then incubated with the secondary antibody (CST, Beverly, MA, USA) for 45 minutes at room temperature. Then, sections were stained using a 3,3′-diaminobenzidine (DAB) procedure, and the nucleus were restained using hematoxylin. Finally, the sections were mounted on slides for image analysis. The percentage of positive staining area was quantified, using Image-Pro Plus 6.0 software (Media Cybernetics, Silver Spring, MD, USA). The expression of protein was detected by mean density.

### 2.5. PAS Staining and Transmission Electron Microscopy

Kidney tissue histopathological examination by the light microscopy with the Periodic Acid-Schiff (PAS) staining was following regular experimental procedures: briefly, slices were deparaffinized and hydrate to water, immersed in 0.5% periodic acid for 15 min, in Schiff's reagent for 30 min, and then in Mayer's haematoxylin solution for 3 min followed by washing in water every step. Finally, after blued up with ammonia solution, slices were used with alcohol and xylene to dehydrate and mount.

Evaluation of kidney injuries was by glomerular sclerosis index and tubulointerstitial injury index. Imaging analysis in PAS staining was carried out as follows: obtained the total area of the glomerulus by checking the enlarged image and then the mesangial area (excluding basement membrane and other parts). The mesangial area index was calculated from the ratio of the mesangial area to the glomerular area expressed by % and quantified by using Image-Pro Plus 6.0 software (Media Cybernetics, Silver Spring, MD, USA). Mesangial matrix index (%) was independently evaluated by three pathologists to assess glomerular damage in an average. The average percentage of mean density area (IOD/area) was quantified using Image-Pro Plus 6.0 software (Media Cybernetics, Silver Spring, MD, USA). Tubular injury was not investigated. For the transmission electron microscopy (TEM) procedure, fresh tissue blocks were isolated by preventing from physical damage with the size of tissue block no more than 1 mm^3^. The tissue blocks were then followed by regular experimental procedures and then cut into ultrathin sections (60-80 nm) with an ultramicrotome. Finally, sections were observed with TEM (Hitachi HT770, Japan). The injured areas were marked by arrows.

### 2.6. Statistical Analysis

All experiments were repeated three times, and data were presented as the mean ± standard error of mean (SEM). Statistical analyses were performed using SPSS 22.0 statistical software (SPSS, Chicago, IL, USA). A one-way analysis of variance (ANOVA) was used to calculate the *P* values, and *P* < 0.05 was considered statistically significant.

## 3. Result

### 3.1. Changes in Body Weight (BW), Kidney Weight/Body Weight (KW/BW) and Levels of Fasting Plasma Glucose and Insulin

After oral pyruvate treatments (1% pyruvate or Pyr-ORS) for 8 weeks (Table 1), the BW of all *db/db* mice was still higher than that of all normal mice, but was significantly lower (*P* < 0.01) in groups Pyr and Pyr-ORS compared with group Con; the KW/BW ratio was higher in group Pyr than that in group Pyr-ORS relative to group Con (*P* < 0.01), indicating that 1.0% pyruvate was more beneficial than 0.35% pyruvate of Pyr-ORS in the BW decrease in diabetic *db/db* mice. Compared with group Con, fasting blood glucose levels were decreased (*P* < 0.05), whereas levels of fasting plasma insulin were significantly increased (*P* < 0.01) in groups Pyr and Pyr-ORS (*P* < 0.01) after the treatment. Results indicated that oral pyruvate in two pyruvate groups almost comparably improved diabetes status in *db/db* mice.

### 3.2. Changes in Serum Cr, BUN, and Cys-C and Levels of Urine Protein and NGAL

In Table 2, compared with group Nor, serum Cr, BUN, and Cys-C, urine NGAL, and 24-hour urinary protein were significantly higher in all *db/db* mice; however, the indicators of early sensitive kidney function, serum Cys-C and urine NGAL, were significantly lower (*P* < 0.05) in groups Pyr and Pyr-ORS after 8 weeks of treatments. In group Pyr, 24-hour urinary protein was also remarkably lower (*P* < 0.05). In terms of serum Cr and BUN, the pyruvate effect was not obvious in the experiment. Data suggested that oral pyruvate in two pyruvate groups comparably protected kidney function against diabetes and delayed DN exacerbation in *db/db* mice.

### 3.3. Changes in Activities of Pyruvate Kinase, LDH, and PDHa/PDHt in Kidney Homogenates

As shown in Table 3, compared to group Con, there were significant alterations (higher or lower) in levels of LDH, PK, and PDHa/PDHt in the kidneys of groups Pyr and Pyr-ORS after the treatments (*P* < 0.05 or *P* < 0.01). Particularly, HG-promoted LDH and HG-inhibited PDHa were mostly recovered. These data strongly showed that oral pyruvate in two pyruvate groups profoundly and comparably improved the key enzymes in glycolysis and glucose oxidative metabolism in diabetic *db/db* mice.

### 3.4. Changes in Sorbitol, Fructose, NAD^+^/NADH Ratio, and AGEs in Kidney Homogenates

As shown in Table 4, compared to group Con, sorbitol, fructose, NAD^+^/NADH ratio, and AGEs in kidney homogenates were markedly altered (higher or lower) in groups Pyr and Pyr-ORS, although sorbitol and fructose were not significantly reduced from statistical study in group Pyr-ORS, after treatments (*P* < 0.05 or *P* < 0.01). Results revealed that oral pyruvate in two pyruvate groups significantly and comparably raised NAD^+^/NADH ratio and ameliorated AGEs formation in kidneys of diabetic *db/db* mice.

### 3.5. Changes in the Expressions of Key Enzyme in Glucometabolic Pathways in Kidney Homogenates

**Figure 1:**
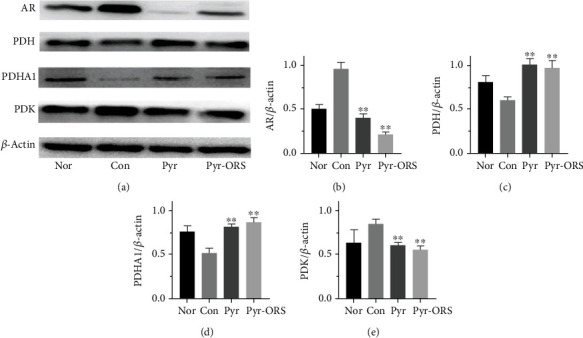
Pyruvate effects on enzyme activities of glucose metabolism in diabetic kidney homogenates after different treatments (*n* = 6). Oral pyruvate comparably increased PDH and PDHA1, but inhibited AK and PDK activities in groups Pyr and Pyr-ORS, compared to group Con, in diabetic *db/db* mice (a). Nor (normal), C57BLKS/J nontreated mice; Con (control), C57BLKS/J diabetic *db/db* nontreated mice; Pyr, *db/db* mice drunk distilled water containing 1% pyruvate; Pyr-ORS, *db/db* mice drunk pyruvate-enriched ORS to replace distilled water, containing 0.35% pyruvate; AR: aldose reductase (b); PDH: pyruvate dehydrogenase (c); PDHA1: PDH E1 component subunit alpha (d); PDK: pyruvate dehydrogenase kinase (e). Values were means ± SEM. ^∗^*P* < 0.05 and ^∗∗^*P* < 0.01 vs. Con.

Figure 1 showed the higher expressions of AR and PDK, but the lower expressions of PDH and PDHA1 (active form of PDH) induced by HG in kidney homogenates in group Con than in group Nor. However, all alterations were reversed after intervention with oral pyruvate in two pyruvate groups. These results strongly suggested that oral pyruvate recovered aberrant sorbitol pathway and reactivated glucose oxidative metabolism, resulting in renoprotection, in diabetic *db/db* mice.

### 3.6. Changes in Indexes of Oxidative Stress and Inflammation in Kidney Homogenates

**Figure 2:**
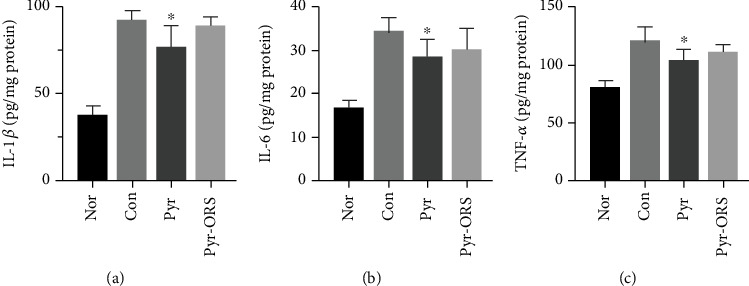
Pyruvate effects on proinflammatory cytokines in diabetic kidney homogenate after different treatments (*n* = 6). Oral pyruvate significantly inhibited IL-1*β*, IL-6, and TNF-*α* in kidney homogenate in group Pyr, compared to group Con, in diabetic *db/db* mice. Nor (normal), C57BLKS/J nontreated mice; Con (control), C57BLKS/J diabetic *db/db* nontreated mice; Pyr, *db/db* mice drunk distilled water containing 1% pyruvate; Pyr-ORS, *db/db* mice drunk pyruvate-enriched ORS to replace distilled water, containing 0.35% pyruvate. (a) Interleukin 1 beta (IL-1*β*), (b) interleukin 6 (IL-6), and (c) tumor necrosis factor-*α* (TNF-*α*). Values represented mean ± SEM, ^∗^*P* < 0.05 vs. Con.

Compared to group Con in Table 5, there were significant changes (higher or lower) in serum ROS, SOD, MDA, CAT, and GSH-PX levels in the kidneys of groups Pyr and Pyr-ORS after the 8-week study period (*P* < 0.05 or *P* < 0.01). In Figure 2, there was a significant decrease in inflammatory factors (IL-1*β*, IL-6, and TNF-*α*) in the kidneys of group Pyr and a slight decrease in group Pyr-ORS after 8 weeks of pyruvate treatments. These data indicated that oral 1.0% or 0.35% pyruvate markedly attenuated oxidative stress: increased SOD, CAT, and GSH-Px, whereas decreased ROS and MDA, with a little inhibition of inflammation in kidneys of diabetic *db/db* mice.

### 3.7. Pyruvate Ameliorated Renal Histopathological Damage

**Figure 3:**
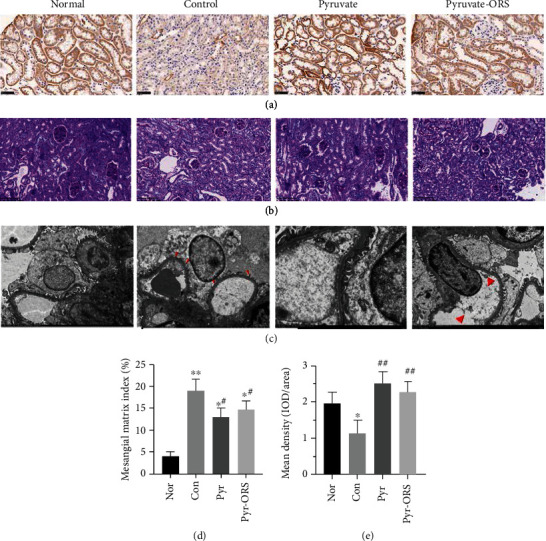
Pyruvate effects on PDH immunohistochemistry in diabetic kidney homogenate and pathological changes in PAS staining and TEM after different treatments (*n* = 6). Oral pyruvate comparably preserved PDH contents with the mean density (IOD/area) in immunohistochemistry staining in groups Pyr and Pyr-ORS, compared with group Con, in diabetic *db/db* mice. Pathological alterations showed reduction of mesangial matrix expansion as well as glomerular volume in groups Pyr and Pyr-ORS relative to group Con in PAS and attenuation of edema in glomerular endothelial cells and podocytes in TEM, as arrows indicated. Nor (normal), C57BLKS/J nontreated mice; Con (control), C57BLKS/J diabetic *db/db* nontreated mice; Pyr, *db/db* mice drunk distilled water containing 1% pyruvate; Pyr-ORS, *db/db* mice drunk pyruvate-enriched ORS to replace distilled water, containing 0.35% pyruvate. The protein expression levels of PDH were evaluated in the mean density (IOD/area) by immunohistochemistry (a). Renal pathological changes were examined by PAS staining and TEM (b, c). Mesangial matrix index (%) and mean density (IOD/area) (d, e). PDH: pyruvate dehydrogenase; IOD: integrated optical density. Values represented mean ± SEM, ^∗^*P* < 0.05 and ^∗∗^*P* < 0.01 vs. Non; ^#^*P* < 0.05 and ^##^*P* < 0.01 vs. Con.

The renoprotective effects of oral pyruvate in ameliorating renal morphological damage were investigated by PAS staining and transmission electron microscope. Immunohistochemistry studies showed that the protein expression levels of PDH by the mean density of integrated optical density (IOD/area) was seriously reduced in group Con, but equally and fully restored in groups Pyr and Pyr-ORS (Figures 3(a) and 3(e)). In Figure 3(b), the pathological changes of kidney tissues in PAS staining were eliminated in groups Pyr and Pyr-ORS in relation to group Con. Compared to the normal mice, there was a higher glomerular volume and mesangial matrix expansion in group Con, but oral pyruvate treatments parallelly attenuated mesangial matrix index and edema of glomerular endothelial cells and podocytes (see arrows in Figure 3(c)) in the kidney microstructure of groups Pyr and Pyr-ORS (Figures 3(b)–3(d)). Results from kidney morphological alterations indicated that oral pyruvate in two pyruvate groups comparably eliminated injuries of kidney histopathology in diabetic *db/db* mice.

## 4. Discussion

### 4.1. Pyruvate Delayed Progression of Diabetes Status and Diabetic Kidney Disease

Present results illustrated that oral pyruvate fluids as drinking water enabled to improve diabetes and attenuate DN progression in diabetic *db/db* mice, a widely acceptable standard model for type 2 diabetic patients. As shown in Tables 1 and 2, pyruvate in both 1% drinking water and Pyr-ORS (0.35% pyruvate) decreased the BW and fasting blood sugar and increased fasting blood insulin in like manner in two pyruvate groups after an 8-week treatment. Oral pyruvate (1% pyruvate or Pyr-ORS) in both groups significantly improved diabetic kidney function as preserved lower levels of blood Cys-C, 24-hour urine protein, and NGAL; further, kidney injuries in mesangial matrix and podocytes (Figures 3(b) and 3(c)) in histopathological alterations were diminished after pyruvate treatments for 8 weeks, compared to group Con. These results demonstrated that oral pyruvate fluids were effective in the treatment of diabetes and DN of *db/db* mice.

Glucometabolic data showed key enzymes: AR in sorbitol pathway, PK in glycolysis, and PDH in glucose oxidative metabolism were profoundly improved (Figures 1(b) and 3(a) and Table 3); oxidative stress and inflammation were reduced by oral pyruvate though inflammatory inhibition was minor in diabetic kidneys of *db/db* mice (Table 5 and Figure 2). Importantly, AGE levels in diabetic kidney were attenuated after oral pyruvate treatment for 8 weeks (Table 4). All findings supported that two oral pyruvate fluids analogously improved diabetic status and delayed DN progression in diabetic *db/db* mice. Notably, prior studies demonstrated that pyruvate improved diabetic ocular complications and discovered that pyruvate inhibited zinc-induced pancreatic islet cell death, and pyruvate supplementation attenuated *β*-cell death *in vitro* and *in vivo* experiments [11, 13, 24, 25]. However, to date, there has been no investigation concerning pyruvate effects on DN. Intriguingly, a preliminary clinical report revealed that a large dose of oral pyruvate (about 1.0 g/kg/d for 7-10 days) apparently controlled diabetes in 6 patients subjected to type 1 diabetes with a tendency of hypoglycaemia. An additional finding showed that oral pyruvate in a large dose improved the insulin secretion of *β*-cells in a mitochondrial DM patient with a reduction of total daily insulin dose [26, 27]. These case reports apparently support the present findings. The diabetic *db/db* mice model used in experiments is basically reflected with diabetic disorders in patients subjected to type 2 diabetes; the promising results here, which consisted with previous experimental findings and the case reports, provided a greater possibility for oral pyruvate to prevent and treat clinical diabetes.

### 4.2. Pyruvate Improved Glucometabolic Disturbances

The following novel findings should be emphasized in pyruvate effects on glucometabolic abnormalities in attenuation of diabetic status and DN.

#### 4.2.1. Pyruvate Improved Glycolytic Pathways in Diabetes

Present data first discovered that aldose reductase (AR) was stimulated in group Con but restored by oral pyruvate treatments in groups Pyr and Pyr-ORS in diabetic *db/db* mice (Figure 1(b)).

**Figure 4:**
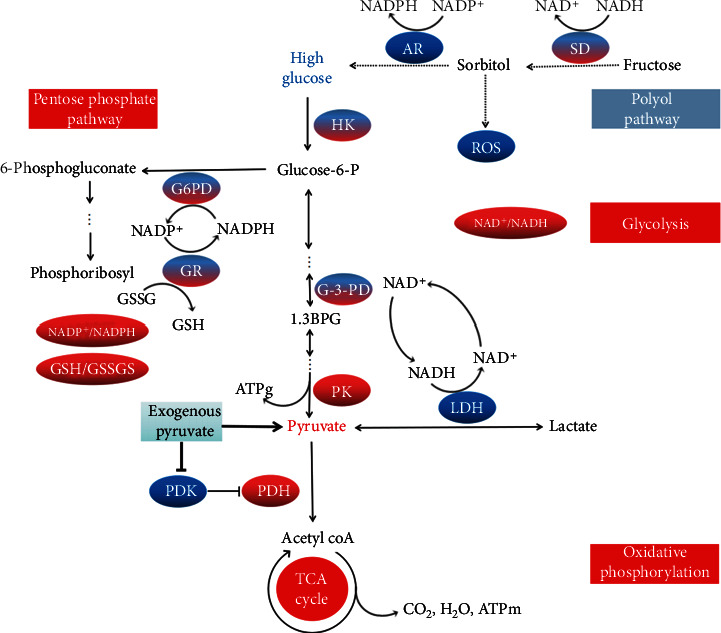
Diagram of the underlying glucometabolic mechanisms on pyruvate protection against diabetic nephropathy. Oral pyruvate enters cytosol via the mitochondrial pyruvate carrier in diabetic *db/db* mice. Pyruvate reductive reaction with LDH reaction couples the NADH oxidation to NAD^+^ with increase of NAD^+^/NADH ratio, prompting glycolytic flux at G-3-PD step. Pyruvate also stimulates PK and inhibits AR activities, leading to inhibit the HG-promoted sorbitol pathway. All restore the inhibited anaerobic glycolytic pathway induced by HG. In mitochondria, pyruvate also reactivates the HG-declined PDH by direct inhibition of PDK, increasing PDHa/PDHt ratio and glucose oxidative phosphorylation in the TCA cycle metabolism, concomitantly reducing the blood glucose level. Oral pyruvate competitive inhibition of AR in sorbitol pathway increases NAD(P)^+^/NAD(P)H ratios. As a result, it restores the suppressed PPP, leading to increase of GSH/GSSG ratios in diabetic kidney. Red color: increase; blue color: inhibition; red/blue color: untested.

It is acknowledged that the AR activity is definitely stimulated in HG and diabetes, contributing to promotion of the sorbitol pathway, additionally due to the hexokinase activity (HK) saturated in hyperglycemia, resulting in NADPH/NADP^+^ and NAD^+^/NADH ratio depletion and subsequent inhibition of the pentose phosphate pathway (PPP), which is the major source of NADPH to sustain GSH [11, 12, 28]. Oral pyruvate declined the promoted AR in diabetic *db/db* mice first supported the previous proposal that pyruvate competitive inhibition of AR in HG conditions [12]. Therefore, hyperglycemia/HG accounts for redox potential inhibition in diabetic tissues and organs (Figure 4). Further, as shown in Table 3, the renal PK activity, one of the key glycolytic enzymes, was also depressed in diabetic *db/db* mice. Anaerobic glycolytic pathway, thus, is suppressed in diabetes, as suggested as so-called pseudohypoxia, which can be significantly attenuated by antioxidant treatments [29, 30], while pyruvate is a well-recognized potent antioxidant. Both HG-induced AR stimulation and PK suppression play critical roles in DN development [3, 30]. However, present findings first comprehensively ascertained that oral pyruvate strengthened canonical glycolytic flux with a decrease of glucose toxic metabolite accumulation by (1) competitive inhibition of AR in sorbitol pathway with a rise of NAD^+^ and NADPH and concomitantly reversing the PPP, leading to GSH/GSSG enhancement, as shown with increases of NAD^+^/NADH and GSH-Px (Tables 4 and 5); (2) reversal of key glycolytic enzymes, such as HK, phosphate fructose kinase (PFK-1), and PK (PK M2 increase may also regulate TCA/mitochondrial pathways [4]), though the 2 formers were not detected; and (3) spontaneously anaerobic pyruvate reduction by the energy free LDH reductive reaction, despite attenuation of HG-raised LDH by pyruvate, coupled with NADH oxidation to NAD^+^, additionally raising the NAD^+^/NADH ratio in diabetes (Table 4), which facilitates glycolysis at the G-3PD step (glyceraldehyde-3-phosphate dehydrogenase), where NAD^+^ is required [30]. Consequently, the canonical anaerobic glycolysis was quite functionally revival in both oral pyruvate-treated groups of diabetic *db/db* mice (Figure 4).

#### 4.2.2. Pyruvate Preserved Oxidative Metabolism in Diabetes

The pyruvate dehydrogenase (PDH) activity plays a critical role in glucose oxidative metabolism. Various pathogenic insults, including critical injuries, such as bleeding, trauma and sepsis, diabetes, aging, and even cancer, may induce the PDH inhibition, leading to specifically distinct metabolic disturbances and acid-base imbalances in various respective insults. The PDH activity is decreased in diabetic animals and humans, and clinical diabetic ketoacidosis even appears more severe PDH inhibition [31, 32]. Data here provided first evidence that oral pyruvate rejuvenated the HG-inhibited PDH activity in diabetes, as shown in Table 3, where renal PDHa/t activities were comparably enhanced in two pyruvate groups. Also, immunohistochemistry of renal PDH activities similarly showed a full restoration in two pyruvate groups in *db/db* mice (Figure 3(a)). Pyruvate, like dichloroacetate (DCA) as a PDH activator [33], conferred a full reactivation of the suppressed PDH in diabetic kidneys of two pyruvate groups, which was consistent with the treatment of hemorrhagic shock and brain trauma [34, 35]. Apparently, the novel finding with oral pyruvate reactivation of PDH along with PK improvement and AR inhibition in diabetic *db/db* mice is of robust significance in clinical treatments of diabetes and diabetic organ complications in the kidney, brain, heart, eye, and skin.

As shown in Figures 1(c)–1(e), PDH and PDHA1 were inhibited, but PDK were prompted in the control group, whereas the above alterations were fully restored in two pyruvate groups. Accordingly, oral pyruvate reversal of PDH was closely associated with its inhibition of HG-enhanced PDK activity in *db/db* mice, as previously demonstrated that pyruvate as mimetic DCA inhibited PDK phosphorylation [36, 37]. By renovation of PDH as well as PC (pyruvate carboxylase) activity induced by pyruvate, oral pyruvate enhanced anaplerosis (replenishment of tricarboxylic acid (TCA)-cycle substrates) and the TCA cycle with sustained oxidative phosphorylation, *in vivo*. Accordingly, the Warburg phenomenon (aerobic glycolysis enhancement with PDH-oxidative phosphorylation inhibition in aerobic conditions) could be reversed. As a result, glucose oxidative metabolism as well as blood sugar was markedly improved, leading to delay diabetic progression in *db/db* mice (Figure 4). In addition, as shown in Table 1, oral pyruvate might stimulate insulin secretion from *β*-cells in diabetic or even nondiabetic patients [26, 27, 38]; thus, enhanced blood insulin might also activate the PDH activity, *in vivo*. Further, the PDH preservation might be additionally contributed with pyruvate antioxidative stress (Table 5). Furthermore, although pyruvate/lactate ratio and ATP generation in blood or kidney were not monitored in the experiment, NAD^+^/NADH ratio was significantly increased in diabetic kidneys following pyruvate treatments (Table 4). It was previously discovered that enteral Pyr-ORS raised blood pyruvate over 5 times with pyruvate/lactate ratio increase and corrected lethal hypoxic lactic acidosis, profoundly enhancing survival in rehydration of rats subjected to severe burn shock [23]; oral pyruvate as drinking water also markedly increased contents of ATP and ATPase in neurons of rats subjected to microgravity for 8 weeks (data submitted for publication).

All in all, present results first demonstrated that oral pyruvate fluids effectively corrected glucometabolic disturbances, particularly by reactivation of glucose oxidative phosphorylation with PDH (PDHa/t) restoration via direct PDK inhibition, in diabetic *db/db* mice. The pyruvate protective effects above against diabetic glucometabolic aberrances, *in vivo*, as AR and PDK inhibition and PDH reactivation were replicated by pyruvate in HK-2 (human renal proximal tubular epithelial) cell lines in HG conditions, *in vitro* (data not shown), which also showed the inhibition of ER stress and cellular apoptosis of HK-2 cells by pyruvate addition in HG [39]. A recent investigation further demonstrated that diabetes markedly inhibited mitochondrial metabolism in pancreatic *β*-cells, whereas early studies displayed pyruvate preservation of islet engrafts function [40, 41]. Present results that showed a consequence of rise in blood insulin levels are in consistence with these findings though islets were not investigated in the experiment.

Therefore, oral pyruvate as a novel strategy, other than current advances with sodium glucose linked transporter-2 (SGLT-2) inhibitors and glucagon-like peptide-1 (GLP-1) receptor agonists [2], treated diabetic status and DN specifically by restoration of regular glucometabolic pathways with promoting glycolysis and glucose oxidative phosphorylation to drive the HG-induced Warburg effect backwards in kidneys of the experimental diabetic *db/db* mice.

#### 4.2.3. Pyruvate Delayed AGE Formation

Another novel finding that has to be noticed is that pyruvate was also shown as an AGE antagonist that parallelly inhibited renal AGEs and oxidative stress levels in two pyruvate groups in *db/db* mice (Tables 4 and 5), though the inflammatory inhibition was not significant with Pyr-ORS in this experimental condition (Figure 2). AGE formation as well as deposition in tissues is an important causative factor of onset and progression in diabetic organ complications, including DN via stimulating oxidative stress, angiotensinogen production, ER stress, and fibrosis in kidney [9, 42, 43]. AGE inhibitors are, at least, partially effective in DN treatments in both animal studies and clinical trials [44]. Recent findings demonstrated that pyruvate was ER stress inhibitable, *in vitro*, via antioxidative stress and antiapoptotic effects and resistant to the AGEs formation, *in vivo*, by a competitive inhibition with glucose and recover of enzyme systems that protect from glycation stress [39, 45]. Pyruvate even showed a significant role in the reversal of AGE formation and attenuated its advancement in diabetic cataract tissues, and its underlying molecular mechanism was also addressed [19, 20, 45]. The oral pyruvate effect on attenuation or prevention of AGE accumulation in diabetes may play a pivotal role in reversing the vicious cycle in DN onset and development.

### 4.3. Pyruvate in ORS Benefits in Clinical Diabetes

Since 2012, Pyr-ORS was first proposed to improve WHO-guided ORS (WHO-ORS I, II or III) that contains bicarbonate or citrate by equimolar pyruvate. Prior studies demonstrate that oral or enteral Pyr-ORS is more beneficial than WHO-ORS in animal shock resuscitation and kidney protection [21–23]. Present data clearly illustrated that oral pyruvate in both Pyr-ORS (0.35% pyruvate) and 1% pyruvate was comparably effective to protect key enzyme activities and histopathological alterations against HG in diabetic mice (Figures 1 and 3).

In clinical settings, a patient drunk 1% pyruvate for 2 liters ingested 20 g of sodium pyruvate, but ingestion of 7-25 g pyruvate has no effectiveness or just limited function in adults due to poor absorption from intestine [46, 47]. Intestinal absorption of sodium salt requires coexisting glucose via Na^+^-glucose cotransporters located in gastrointestinal epithelium in mammalians [21–23]. In contrast, 1% pyruvate is effective in diabetic rodents, due to feed pyruvate always in Na^+^-glucose fluid with drinking and eating in animals. Given the findings that two pyruvate fluids showed a basically equal effect, 0.35% pyruvate with 1.35% glucose in Pyr-ORS may be beneficial and practicable to prevent and treat diabetes in humans. Nevertheless, it is also worthy to note that oral Pyr-ORS here just delayed the DN progression, rather than fully prohibited the DN development in *db/db* mice. The pyruvate dosage might be not optimal; the dose-effect test and a long-term investigation are required. Further studies with a large animal group size in various models are essential to verify oral pyruvate (Pyr-ORS) effects on diabetes and DN and its effects on the protection of diabetic pancreatic *β*-cells. Because several clinical studies in various diseases treated with large doses (around 1.0 g/kg) of pyruvate products at the time reported that oral or systemic pyruvate administration indicated the clinical effectiveness and tolerance without adverse effects [26, 27, 38, 48–50], clinical trials are safe and urgently warranted in diabetic patients with a Pyr-ORS formula modified if needed.

Finally, it is hereby notable that ethyl pyruvate (EP, a derivative of pyruvate sodium salt) has been reported for the potential intervention of diabetes and DN [51], which further strengthens pyruvate effects on diabetes. However, there is a distinct difference between EP and sodium pyruvate: EP is helpful in animals, but not in humans; the failure of a phase II clinical trial on EP may support the hypothesis, making its clinical prospective hopelessly [52, 53].

Taking together, results here suggest that oral Pyr-ORS may provide a novel strategy in pharmacologic interventions of clinical diabetes and its organ complications, even diabetic ketoacidosis and may be a novel therapeutic approach, even as a functional drink, in the prevention and treatment of diabetes and DN in a large population.

## 5. Conclusion

Oral Pyr-ORS has a comparable effectiveness with 1% pyruvate in the treatment of diabetes and DN development in diabetic *db/db* mice. Oral pyruvate may renovate glucometabolic profile, particularly inhibit HG-promoted AR activity, and reactivate HG-depressed PK and PDH, via inhibition of PDK, activities to reverse the Warburg effect in diabetic glucometabolic defects. In addition, oral pyruvate may exert antioxidative stress and inhibit AGE formation. Consequently, oral pyruvate may turn the vicious circle to a virtuous cycle of glucometabolic disorders in diabetes and DN. Further study is required particularly in the dose-response manner and long-term investigations with a large group size. A novel therapeutic strategy with Pyr-ORS may be beneficial in clinical prevention and treatment of type 2 diabetes and DN.

## Figures and Tables

**Table 1:** Changes in BW, KW/BW, blood glucose, and insulin levels in diabetic mice after different treatments (*n* = 6).

	Nor	Con	Pyr	Pyr-ORS
BW (g)				
Week 0	21.2 ± 2.3	43.8 ± 1.5^∗∗^	45.9 ± 1.8^##^	43.8 ± 1.30^##^
Week 8	24.8 ± 1.3	63.0 ± 4.0^∗∗^	49.1 ± 6.5^∗∗^^##^	51.6 ± 4.0^∗∗^^##^
KW/BW (mg/g)				
Week 0	—	—	—	—
Week 8	16.1 ± 1.9	7.8 ± 0.9^∗∗^	11.2 ± 2.2^∗∗^^##^	9.7 ± 0.4^∗∗^
Glucose (mmol/l)				
Week 0	5.70 ± 1.59	21.0 ± 2.7^∗∗^	23.6 ± 3.2^∗∗^	20.1 ± 1.5^∗∗^
Week 8	6.6 ± 0.4	26.8 ± 3.1^∗∗^	18.5 ± 6.6^∗∗^^#^	19.0 ± 5.0^∗∗^^#^
Insulin (ng/ml)				
Week 0	3.5 ± 1.2	5.3 ± 1.4^∗^	5.2 ± 1.3^∗^	5.8 ± 1.4^∗^
Week 8	3.3 ± 1.1	5.4 ± 1.7^∗^	12.6 ± 1.4^∗∗^^##^	11.4 ± 2.1^∗∗^^##^

Nor (normal), C57BLKS/J nontreated mice; Con (control), C57BLKS/J diabetic *db/db* nontreated mice; Pyr, *db/db* mice drunk distilled water containing 1% pyruvate; Pyr-ORS, *db/db* mice drunk pyruvate-enriched ORS to replace distilled water, containing 0.35% pyruvate; BW: body weight; KW/BW: kidney weight/body weight; glucose: fasting plasma glucose; insulin: fasting plasma insulin; ^∗^*P* < 0.05 and ^∗∗^*P* < 0.01 vs. Nor; ^#^*P* < 0.05 and ^##^*P* < 0.01 vs. Con. Values were means ± SEM at week 0 and at week 8, respectively.

**Table 2:** Changes in Serum Cr, BUN, and Cys-C and levels of urine protein and NGAL after different treatments (*n* = 6).

	Nor	Con	Pyr	Pyr-ORS
Serum creatinine (mg/dl)				
Week 0	0.15 ± 0.057	0.25 ± 0.049^∗^	0.27 ± 0.055^∗^	0.26 ± 0.071^∗^
Week 8	0.19 ± 0.44	1.11 ± 0.12^∗∗^	0.66 ± 0.15^∗∗^^#^	1.00 ± 0.20^∗∗^
Serum BUN (mg/dl)				
Week 0	12.9 ± 1.9	20.5 ± 4.3^∗^	20.5 ± 4.3^∗^	25.5 ± 5.3^∗∗^
Week 8	15.1 ± 2.9	57.1 ± 5.3^∗∗^	57.1 ± 5.3^∗∗^	52.4 ± 5.2^∗∗^
Serum Cystatin C (ng/ml)				
Week 0	0.7 ± 0.6	2.2 ± 0.5^∗∗^	2.1 ± 0.5^∗∗^	2.1 ± 0.4^∗∗^
Week 8	0.7 ± 0.7	3.5 ± 0.5^∗∗^	2.7 ± 0.3^∗∗^^#^	2.6 ± 0.6^∗∗^^#^
Urine NGAL (ng/24 h)				
Week 0	63.6 ± 13.5	318.0 ± 10.4^∗∗^	325.5 ± 4.5^∗∗^	322.7 ± 10.6^∗∗^
Week 8	66.3 ± 11.0	544.5 ± 40.5^∗∗^	355.8 ± 32.13^∗∗^^##^	348.28 ± 28.8^∗∗^^##^
24-hour urinary protein (mg)				
Week 0	0.15 ± 0.057	0.25 ± 0.049^∗^	0.27 ± 0.055^∗^	0.26 ± 0.071^∗^
Week 8	0.19 ± 0.44	1.11 ± 0.12^∗∗^	0.66 ± 0.15^∗∗^^#^	1.00 ± 0.20^∗∗^

Nor (normal), C57BLKS/J nontreated mice; Con (control), C57BLKS/J diabetic *db/db* nontreated mice; Pyr, *db/db* mice drunk distilled water containing 1% pyruvate; Pyr-ORS, *db/db* mice drunk pyruvate-enriched ORS to replace distilled water, containing 0.35% pyruvate; BUN: blood urea nitrogen; NAGL: neutrophil gelatinase-associated lipocalin. ^∗^*P* < 0.05 and ^∗∗^*P* < 0.01 vs. Nor; ^#^*P* < 0.05 and ^##^*P* < 0.01 vs. Con. Values were means ± SEM at week 0 and at week 8, respectively.

**Table 3:** Changes in LDH, PK, and PDHa/PDHt in kidney homogenates after different treatments (*n* = 6).

	Nor	Con	Pyr	Pyr-ORS
LDH (mU/mg protein)				
Week 0	—	—	—	—
Week 8	10.9 ± 3.1	45.5 ± 4.0^∗∗^	25.1 ± 6.9^∗^^##^	28.6 ± 8.8^∗∗^^##^
PK (mU/mg protein)				
Week 0	—	—	—	—
Week 8	18.1 ± 2.3	9.6 ± 1.6^∗∗^	12.6 ± 1.4^∗∗^^##^	11.7 ± 1.3^∗∗^^#^
PDHa/PDHt ratio				
Week 0	—	—	—	—
Week 8	78.6 ± 6.4	54.3 ± 6.3^∗∗^	70.2 ± 11.5^#^	67.5 ± 8.7^#^

Nor (normal), C57BLKS/J nontreated mice; Con (control), C57BLKS/J diabetic *db/db* nontreated mice; Pyr, *db/db* mice drunk distilled water containing 1% pyruvate; Pyr-ORS, *db/db* mice drunk pyruvate-enriched ORS to replace distilled water, containing 0.35% pyruvate; LDH: lactate dehydrogenase; PK: pyruvate kinase; PDHa/PDHt: activities of pyruvate dehydrogenase/total pyruvate dehydrogenase. ^∗^*P* < 0.05 and ^∗∗^*P* < 0.01 vs. Nor; ^#^*P* < 0.05 and ^##^*P* < 0.01 vs. Con. Values were means ± SEM at week 0 and at week 8, respectively.

**Table 4:** Changes in sorbitol, fructose, NAD^+^/NADH ratio, and AGEs in kidney homogenates after different treatments (*n* = 6).

	Nor	Con	Pyr	Pyr-ORS
Sorbitol (mmol/mg protein)				
Week 0	—	—	—	—
Week 8	3.2 ± 0.5	8.2 ± 0.7^∗∗^	6.7 ± 1.0^∗∗^^#^	7.4 ± 0.9^∗∗^
Fructose (mmol/mg protein)				
Week 0	—	—	—	—
Week 8	2.9 ± 0.4	3.9 ± 0.4^∗∗^	2.9 ± 0.2^∗∗^^##^	3.5 ± 0.5^∗^
NAD^+^/NADH ratio				
Week 0	—	—	—	—
Week 8	1.8 ± 0.3	0.85 ± 0.1^∗∗^	1.3 ± 0.1^∗^^##^	1.2 ± 0.1^∗^^##^
AGEs (U/mg protein)				
Week 0	—	—	—	—
Week 8	297.8 ± 26.0	575.5 ± 84.9^∗∗^	379.1 + 28.4^∗^^##^	387.0 ± 43.3^∗^^##^

Nor (normal), C57BLKS/J nontreated mice; Con (control), C57BLKS/J diabetic *db/db* nontreated mice; Pyr, *db/db* mice drunk distilled water containing 1% pyruvate; Pyr-ORS, *db/db* mice drunk pyruvate-enriched ORS to replace distilled water, containing 0.35% pyruvate; AGEs: advanced glycation end products; NAD^+^/NADH: nicotinamide adenine dinucleotide (oxidized form/reduced form). ^∗^*P* < 0.05 and ^∗∗^*P* < 0.01 vs. Nor; ^#^*P* < 0.05 and ^##^*P* < 0.01 vs. Con. Values were means ± SEM at week 0 and at week 8, respectively.

**Table 5:** Changes in ROS, SOD, CAT, MDA, and GSH-PX in kidney homogenates after different treatments (*n* = 6).

	Nor	Con	Pyr	Pyr-ORS
ROS (IU/mg protein)				
Week 0	331.0 ± 18.2	388.0 ± 29.0^∗^	365.6 ± 16.9^∗^	381.0 ± 19.2^∗^
Week 8	338.4 ± 33.0	410.9 ± 25.5^∗^	301.5 ± 30.6^#^	329.8 ± 64.2^#^
SOD (U/mg protein)				
Week 0	—	—	—	—
Week 8	60.4 ± 3.5	30.2 ± 1.8^∗∗^	39.3 ± 2.5^∗∗^^##^	35.3 ± 3.0^∗∗^^#^
CAT (U/mg protein)				
Week 0	—	—	—	—
Week 8	25.5 ± 1.13	17.7 ± 1.4^∗∗^	20.4 ± 0.6^∗∗^^#^	20.8 ± 3.0^∗∗^^##^
MDA (*μ*mol/mg protein)				
Week 0	13.3 ± 2.4	17.0 ± 2.5^∗^	18.6 ± 1.8^∗∗^	17.4 ± 1.7^∗^
Week 8	17.4 ± 2.1	28.2 ± 5.2^∗∗^	21.8 ± 2.7^#^	20.6 ± 2.0^#^
GSH-PX (*μ*g/g protein)				
Week 0	—	—	—	—
Week 8	339.2 ± 21.4	242.2 ± 24.03^∗∗^	305.3 ± 12.0^∗∗^^##^	286.4 ± 18.4^∗∗^^#^

Nor (normal), C57BLKS/J nontreated mice; Con (control), C57BLKS/J diabetic *db/db* nontreated mice; Pyr, *db/db* mice drunk distilled water containing 1% pyruvate; Pyr-ORS, *db/db* mice drunk pyruvate-enriched ORS to replace distilled water, containing 0.35% pyruvate; ROS: radical oxygen species; SOD: superoxide dismutase; CAT: catalase; MDA: malondialdehyde. ^∗^*P* < 0.05 and ^∗∗^*P* < 0.01 vs. Nor; ^#^*P* < 0.05 and ^##^*P* < 0.01 vs. Con. Values were means ± SEM at week 0 and at week 8, respectively.

## Data Availability

Data can be provided upon requests.

## Abbreviations

AGEs: Advanced glycation end products
AR: Aldose reductase
Cyst C: Cystatin C
DM: Diabetes mellitus
DN: Diabetic nephropathy
ER: Endoplasmic reticulum
GSH-Px: Glutathione peroxidase
HG: High glucose
LDH: Lactate dehydrogenase
NAD(P)^+^/NAD(P)H: Nicotinamide adenine dinucleotide (phosphate) (oxidized/reduced form)
NGAL: Neutrophil gelatinase-associated lipocalin
ORS: Oral rehydration solution
GSH/GSSG: Glutathione (reduced/oxidized form)
PDH: Pyruvate dehydrogenase
Pyr-ORS: Pyruvate-ORS
PDHa/t: PDHactive/total
PDHA1: Active form of PDH
PDK: Pyruvate dehydrogenase kinase
PK: Pyruvate kinase
PPP: Pentose phosphate pathway
ROS: Radical oxygen species
SOD: Superoxide dismutase
TCA cycle: Tricarboxylic acid cycle.
