# Correction to “Pyruvate-Enriched Oral Rehydration Solution Improves Glucometabolic Disorders in the Kidneys of Diabetic *db/db* Mice”

**DOI:** 10.1155/jdr/9806272

**Published:** 2025-10-22

X. M. Zhang, H. Deng, J. D. Tong, et al., “Pyruvate-Enriched Oral Rehydration Solution Improves Glucometabolic Disorders in the Kidneys of Diabetic *db/db* Mice,” *Journal of Diabetes Research* 2020 (2020): 2817972, https://doi.org/10.1155/2020/2817972.

In the article titled “Pyruvate-Enriched Oral Rehydration Solution Improves Glucometabolic Disorders in the Kidneys of Diabetic *db/db* Mice,” an error occurred in Reference 48.

The reference was incorrectly listed as:

48. M. L. Krinsky and M. D. Topazian, “A Novel Approach to Common Bile Duct Stone Extraction,” *Gastrointestinal Endoscopy*, vol. 46, no. 4, pp. 382-383, 1997.

It should read as:

48. L. Mateva, I. Petkova, K. Petrov, et al., “Ten-Day Course of Sodium Pyruvate Infusions in Patients With Chronic Liver Diseases (CLD),” *Japanese Pharmacology and Therapy*, vol. 24, no. 12, pp. 2629-2639,1996.

We apologize for this error.

